# Potential of Carbon Sequestration in Biominerals of *Buglossoides arvensis* (L.) I.M. Johnst. Fruits Under Contrasting Soil Calcium Content

**DOI:** 10.3390/plants15131940

**Published:** 2026-06-24

**Authors:** Elena Ikkonen, Elizaveta Linkevich, Ksenia Nikerova

**Affiliations:** 1 Department of Multidisciplinary Scientific Research, Karelian Research Center of the Russian Academy of Sciences, 185910 Petrozavodsk, Russia; maltseva2@gmail.com; 2 Department of General Chemistry, Institute of Biology, Ecology and Agricultural Technologies, Petrozavodsk State University, 185035 Petrozavodsk, Russia; knikerova@yandex.ru

**Keywords:** biomineralization, phytoliths, carbonate fraction, fruit yield, occluded carbon

## Abstract

Biomineralization in plant tissues is a widespread process accompanied by carbon fixation in biogenic minerals. This study aimed to evaluate the effect of CaCO_3_ application to soil on the formation and localization of biominerals in the pericarp of fruits of *Buglossoides arvensis* (L.) I.M. Johnst., as well as on the accumulation of carbon in minerals. *B. arvensis* seeds were sown in the soil treated with CaCO_3_ at concentrations of 0.0 (0 Ca), 2.5 (2.5 Ca), 5.0 (5 Ca), 7.5 (7.5 Ca), and 10.0 (10 Ca) t ha^−1^. As a result of CaCO_3_ application, on average across all treatments, the increase in soil pH was 30%, and the calcium and silicon content in the soil increased by 60 and 39%, respectively. The fruit weight was 4, 28, 42, and 21% higher in 2.5 Ca, 5 Ca, 7.5 Ca, and 10 Ca plants than in 0 Ca plants. Scanning electron microscopy analysis revealed the presence of silica and calcium carbonate in the pericarp of *B. arvensis* fruits, but showed no significant differences in the localization of biominerals in the pericarps between the treatments. The content of biosilica (phytoliths) was lower in 2.5 Ca, 5 Ca, 7.5 Ca, and 10 Ca plants than in 0 Ca plants, respectively, by 11, 14, 25, and 19%. The content of organic carbon occluded in a unit mass of phytoliths was, on average, 49% higher in treated than in 0 Ca plants. The content of carbonate fraction in fruits was 13, 14, 20, and 21% higher in 2.5 Ca, 5 Ca, 7.5 Ca, and 10 Ca plants than in 0 Ca plants, reflecting the effect of soil calcium levels on carbonate content in *B. arvensis* pericarp. Thus, in the pericarp of fruits, the ratio of silica to carbonates changed towards a decrease in silica content and an increase in carbonate content as the availability of calcium in the soil increased. In summary, *B. arvensis* responds to increased soil calcium and soil pH by increasing carbon accumulation in biominerals formed in fruit pericarps, supporting the potential for variability in plant biomineralization characteristics under changing growth conditions.

## 1. Introduction

The formation of minerals by plants is a physiologically significant process that occurs in various organs of plants of different families [1,2]. Calcium and silica precipitation are the most common forms of biomineralisation in plants, as evidenced by numerous studies [3,4,5,6,7]. The physiological significance of biomineral formation may lie in the removal of excess elements absorbed in quantities exceeding the needs of plant metabolism, as well as in ensuring the mechanical strength of tissues, protecting plants from temperature, water, salt, and radiation stress, as well as from pathogens, herbivores, etc. [2,5].

The presence of mineral structures in the tissues of various plant organs, including seeds or fruits, has been established [8,9]. As Ostroumova and Zakharova noted, in representatives of the Umbelliferae family, mineralization is manifested in all layers of the pericarp [8]. Despite differences in morphological characteristics, fruits of species of the tribe Lithospermeae (Boraginaceae family) share a common feature: a hard pericarp, the hardness of which is due to the deposition of biogenic minerals [9,10,11]. The mineralized pericarp can protect the embryo from water and mineral losses, radiation, and temperature fluctuations, as well as from soil insects and pathogens.

It has recently been shown that in the fruits of *Buglossoides arvensis* (L.) I.M. Johnst. belonging to the tribe Lithospermeae, the pericarp is completely mineralized with deposits of amorphous silica (SiO_2_) and calcium carbonate (CaCO_3_) in the form of calcite [12]. The authors showed that the deposition of silica and calcite throughout the entire thickness of the *B. arvensis* pericarp is strictly differentiated between cellular structures with a distinct pattern. The cell walls were found to be mineralized with silica, and the intracellular and pore spaces contained high concentrations of calcium, reflecting their biomineralization by calcite [12].

In scientific literature, silica particles formed in living plant tissues are described as phytoliths [13]. Regardless of the type of silica precipitation, phytoliths show organic matter occlusions within their structures [14]. Phytolith organic carbon, referred to in the scientific literature as phytolith-occluded carbon (PhytOC), is present as oxidized forms of cellulose, lignin, and carboxylic acids [15] and is absorbed by plants from the atmosphere to a much greater extent than from a soil [9,10,14,16,17]. Previous studies reported that phytoliths occlude 0.2–5.8% of organic C for most plants studied [16]. PhytOC, as well as plant biocarbonate carbon, being more resistant to degradation than other components of soil organic carbon, represents a more stable form of fixed soil carbon. Thus, the formation of biominerals in plants can be considered as one of the biogeochemical carbon fixation mechanisms [18]. Although carbon occluded by phytoliths in various terrestrial ecosystems is currently being actively studied [14,16,19,20,21,22], little is known about carbon accumulation in the carbonate fraction, especially for the Boraginaceae family.

In addition to the fact that different plant species vary in the amount of phytoliths formed [16], a few studies have shown that soil conditions can affect mineral formation in plants [23,24]. In a pot experiment, Li et al. [23] found that although combined silicon-phosphorus fertilization did not affect the PhytOC content, it increased the phytolith content in rice. Interestingly, increasing the phosphorus application rate can reduce phytolith formation in plants [23]. Sun et al. [24] showed that the occurrence of phytoliths in the stem, leaf, and sheath of rice was higher in Si-rich than Si-poor soil. Despite these advances, our knowledge of the variability of biomineral content in plants under environmental conditions and the contribution of mineral formation to global carbon sequestration remains limited.

This study examined the presence of variability in biomineral formation and their carbon occlusion in *B. arvensis* fruits under different soil calcium content. *B. arvensis* is a weed, undemanding of nutrients, but prefers neutral or alkaline soils. This species is not found in the study area, probably due to the high soil acidity typical for this region (northern Europe, Karelia). We hypothesized that low calcium content and soil acidity negatively affect the ability of plants to form amorphous silica and calcite in fruits, resulting in only partial, rather than complete, mineralization of the pericarp. We also hypothesized that increasing soil calcium and alkalinity would not only promote continuous pericarp mineralization but also increase the incorporation of carbon into biominerals.

To estimate the carbon stock in fruit biominerals, we determined fruit yield and the main processes responsible for yield formation, such as growth, development, photosynthesis, and leaf respiration. Besides *B. arvensis*, the formation of amorphous silica and carbonates has been found in other members of the Boraginaceae family [11,12,25], but *B. arvensis* was chosen for this study because it is a fast-growing annual species with a short growing season. *B. arvensis* is a native species of Eurasia, invasive throughout much of North America. This weedy species grows in arable lands, shrublands, forests, and synanthropic ecosystems. Typically, the fruits of this species contain four nutlets.

## 2. Results

### 2.1. Soil Properties

Among all the soil characteristics studied, CaCO_3_ application had the greatest effect on soil acidity, as well as Ca and Si concentrations (Table 1). Among all treatments, the soil Ca and Si contents were lowest in the 0 Ca treatment and highest in the 10 Ca treatment. Total C and N content were lowest in the 10 Ca treatment and highest in the 5 Ca treatment. Total and available P were highest in the 0 Ca treatment. Soil K content increased with the addition of CaCO_3_, regardless of the application rate. We suggest that the variability in soil C, N, and P is likely due to pre-existing soil heterogeneity and does not reflect the effect of CaCO_3_ application on these soil characteristics. We also assume that the observed differences in the contents of these macronutrients between treatments were not significant enough to affect plant growth and mineralization processes in the weedy species *B. arvensis*.

### 2.2. Buglossoides Arvensis Growth Parameters

Under the conditions of the European North, the seedlings of *B. arvensis* appeared in the third week of April 2025. The seedlings quickly accumulated biomass, and the beginning of flowering of the plants was noted in early June. By the beginning of August, the plants had shed their leaves, and the stems had dried out.

Although calcium application did not affect the growth phases of plants, it significantly affected seedling germination, with the addition of CaCO_3_ leading to improved germination (Table 2). Soil treatment resulted in an increase in the plant height and shoot biomass accumulation. It should be noted that the highest values of these parameters were achieved not at the highest dose of CaCO_3_ used in the study (10 t ha^−1^) but at lower doses such as 5.0 and 7.5 t ha^−1^. The 10 Ca plants had the highest, and the 0 Ca plants had the lowest nutlet mass, with no differences in this parameter between 2.5 Ca, 5 Ca, and 7.5 Ca plants. During the experiment, no cases of seedling mortality were recorded in any of the treatments. No significant differences in pericarp weight or inflorescence number in *B. arvensis* plants were found between the treatments. Calcium addition increased the number of fruits and nutlets per plant, as well as crop yield, whether calculated in g plant^–1^ or t ha^–1^.

### 2.3. Buglossoides Arvensis Physiological Traits

The study did not reveal any significant effect of CaCO_3_ application on the main physiological traits of *B. arvensis*, such as net CO_2_ assimilation rate, transpiration, stomatal opening, as well as leaf respiration during the measurement period (Table 3).

### 2.4. Nutlet Morphology

**Figure 1:**
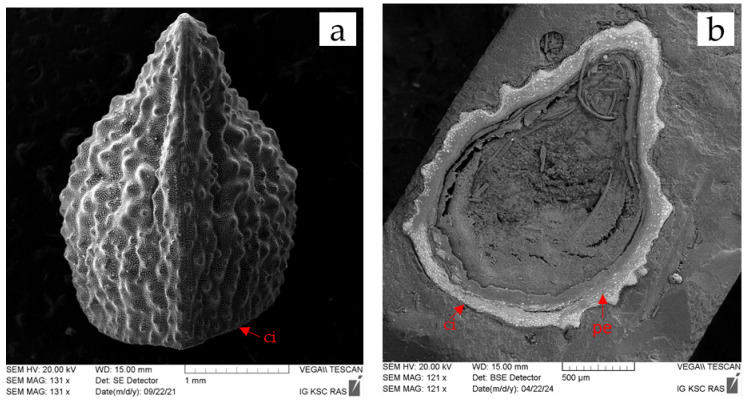
Secondary electron images of intact nutlet (**a**) and nutlet cross-sections (**b**) of *Buglossoides arvensis* fruit. ‘ci’ means cicatrix, ‘pe’ means pericarp. The scale bar is 1 mm for panel (**a**) and 500 µm for panel (**b**).

Among all the treatments, the average nutlet height achieved 2.15 mm (Figure 1a, Table 4). While calcium application did not affect nutlet width, the highest nutlet height was recorded in 2.5 Ca plants, but no significant differences in this parameter were found between 0 Ca, 5 Ca, 7.5 Ca, and 10 Ca plants. Nutlet cross-sections demonstrated high variability in pericarp thickness along the entire length from the apex of the nutlet to the cicatrix, regardless of the intensity of soil treatment with CaCO_3_ (Figure 1b). Soil Ca did not have a significant effect on pericarpal thickness in the cicatrix area, but outside the cicatrix, the thickness of the pericarp was greatest in fruits of 2.5 Ca and 10 Ca plants and least in fruits of 0 Ca and 7.5 Ca plants (Table 4).

### 2.5. SEM Analysis of Nutlet Pericarp

**Figure 2:**
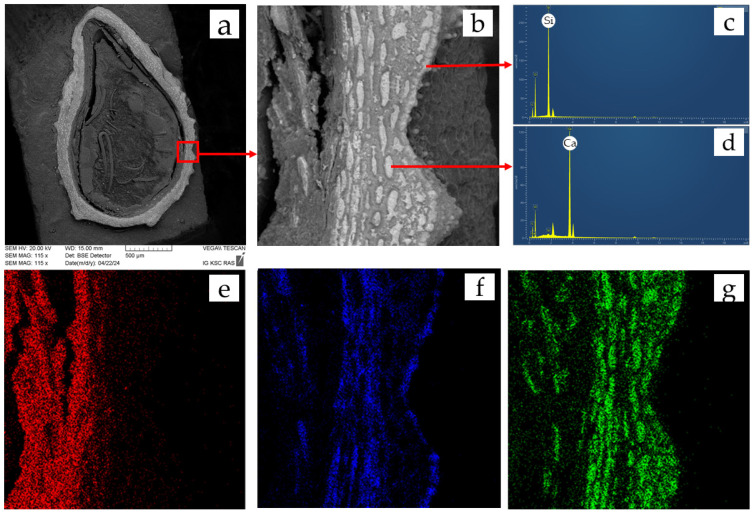
Representative backscatter scanning electron images of nutlet cross-section (**a**), cross-section fragment (**b**), EDS spectra (**c**,**d**), as well as C (**e**), Si (**f**), and Ca (**g**) content in the pericarp of *Buglossoides arvensis*. Colors indicate C in red, Si in blue, and Ca in green.

SEM images and SEM-based EDX analyses of nutlet cross-sections revealed heterogeneity in the elemental composition of the pericarp of plants of all treatments (Figure 2). The chemical composition of structures varied from high Si concentration structures (Figure 2c) to high Ca structures (Figure 2d). A recent study based on the results of X-ray powder diffraction analysis, energy-dispersive X-ray analysis, Raman spectroscopy, and elemental mapping using an electron backscatter (BSE) detector has proven the formation of amorphous silica and calcium carbonate in the form of calcite in the pericarp of *B.* arvensis [12]. Since the BSE images and EDX analysis results in our study are largely consistent with those of the recent study [12], the high silicon and calcium concentrations most likely reflect the presence of the same mineral phases, silica and calcium carbonate, in the fruits studied. Element mapping showed the distribution of elements with C in red, Si in blue, and Ca in green (Figure 2e–g). Visualization of the chemical composition did not reveal any differences in the distribution of mineral phases in the pericarp between the treatments. A common feature for the nutlets of all treated plants was the occurrence of mineral phases throughout the entire thickness of the pericarp, high Si content in the outer wall of epidermal cells, visualization of high Si contents in the cell walls, and high Ca concentrations in intracellular and pore space.

Normalized EDS data of SEM-based EDX analyses showed the presence of carbon in pericarps of all treatments. Although the normalized EDS data were qualitative rather than quantitative, they showed that CaCO_3_ application tended to increase pericarp calcium content, with no differences observed between the Ca-treated plants.

### 2.6. Carbon Content in Biominerals of Buglossoides arvensis Fruits

**Figure 3:**
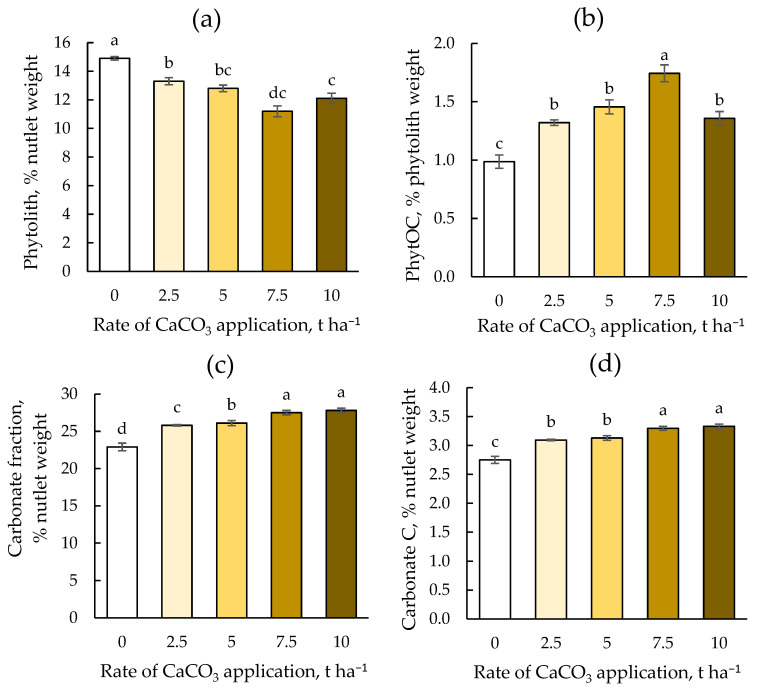
Content of phytolith (**a**), phytolith-occluded carbon (PhytOC, (**b**)), carbonate fraction (**c**), and carbonate carbon (**d**) content in nutlets of *Buglossoides arvensis* grown on soil treated with CaCO_3_ at concentrations of 0.0 (0), 2.5 (2.5), 5.0 (5), 7.5 (7.5), and 10.0 (10) t ha^−1^. Different letters indicate significant differences between the means.

The content of biosilica (phytoliths) in *B. arvensis* fruits varied from 11 to 15% of the nutlet weight (Figure 3a). An increase in the concentration of calcium in the soil led to a decrease in the content of phytoliths in fruits. The occluded carbon (PhytOC) content ranged from around 1.0 to 1.7% (Figure 3b). The 7.5 Ca plants had the highest, and the 0 Ca plants had the lowest PhytOC content, with no significant differences in parameter values between 2.5 Ca, 5 Ca, and 10 Ca plants. Among all treatments, the average content of carbonate fraction in nutlets was found to achieve 26%, with an increase in the content observed as the CaCO_3_ application rate increased (Figure 3c). According to the lime-mediated increase in carbonate fraction content, the carbon content of fruit carbonates per nutlet increased (Figure 3d).

**Figure 4:**
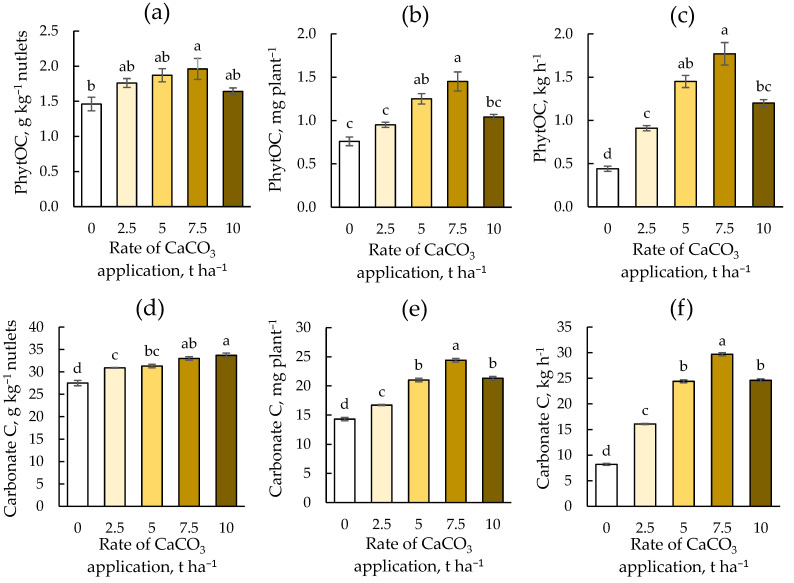
PhytOC content per kilogram of nutlets (**a**), plant (**b**), hectare (**c**), and carbonate C content per kilogram of nutlets (**d**), plant (**e**), hectare (**f**) in fruits of *Buglossoides arvensis* grown on soil treated with CaCO_3_ at concentrations of 0.0 (0), 2.5 (2.5), 5.0 (5), 7.5 (7.5), and 10.0 (10) t ha**^−^**^1^. Different letters indicate significant differences between the means.

The PhytOC content per kilogram of nutlets, per plant, or per hectare was the highest in 7.5 Ca plants and lowest in 0 Ca plants (Figure 4a–c). A tendency for these parameters to increase with increasing soil Ca level was observed only in the 0 Ca–7.5 Ca treatment range. 10 Ca plants showed a decrease in carbon occlusion in phytoliths relative to 7.5 Ca plants. According to the increase in soil Ca, the carbonate C content per kilogram of nutlets tended to increase across the entire range of CaCO_3_ doses used in the study (Figure 4d). Carbonate content of *B. arvensis* fruits, calculated per plant or per hectare, increased from the 0 Ca to the 7.5 Ca treatment, but decreased in 10 Ca plants compared to 7.5 Ca plants (Figure 4e,f).

## 3. Discussion

Recently, the scientific community has paid attention to the ecological role of biomineralization in plants, which involves removing carbon from the atmospheric cycle and sequestering it in the form of soil minerals [2,16]. Thus, studying carbon sequestration in the form of biominerals in plants may make a contribution to the assessment of biogeochemical carbon sinks. In our study, we sought to quantify the potential ability of a representative of the Boraginaceae family to form biominerals in fruits and fix carbon in them, as well as to recognize the variability of this plant’s ability depending on soil properties, in particular, the availability of calcium to plants.

The CaCO_3_ application affected not only the availability of Ca to *B. arvensis*, but also the content of mobile K and Si in the soil (Table 1). Increased content of soil K and Si is most likely due to increased soil pH under lime application. A strong positive correlation between extractable soil Si and soil pH was documented in numerous studies [26,27,28]. Hydrolysis of calcium salts enhances the release of hydroxyl groups, which makes Si more mobile [26]. Thus, liming of non-carbonate soils leads to an increase in the availability of Si for plants [27], which was also confirmed by the results of our study.

CaCO_3_ application to the soil had a positive effect on *B. arvensis* growth parameters (Table 2). The importance of calcium in plant biological processes is well known, highlighting its critical role in plant growth and development [29]. In addition to stimulating plant growth, calcium acts as a primary neutralizer of soil acidity, reducing toxic hydrogen ions and raising soil pH [30]. The use of calcium-containing fertilizers such as lime replaces acidic cations in soil colloids, increasing base saturation. Soil pH plays a vital role in plant growth [31] as it affects a number of biological and physicochemical processes in the soil related to plant nutrient availability [32]. Our study also revealed that soil liming improved *B. arvensis* germination, plant biomass accumulation, fruit weight, and number, which consequently increased both per plant and per area yield. However, CaCO_3_ application did not affect the timing of the onset of main phenological phases of *B. arvensis*, or the rate of main physiological processes such as photosynthesis, respiration, and water exchange in plants (Table 3). Apparently, other plant mechanisms were largely responsible for the increase in *B. arvensis* fruit yield under increased soil Ca content.

Increased soil Ca content could be responsible for an increase in the carbonate content in *B. arvensis* fruits, although this increase was not monotonic at all application rates studied (Figure 3 and Figure 4). The occurrence of high Ca content (Figure 2) indicated the presence of calcium carbonate, which confirms the previously identified deposition of this mineral in the form of calcite in the fruits of *B. arvensis* [9,12]. It has previously been suggested that CaCO_3_ formed in the fruits of *B. arvensis* is accumulated from carbon formed during photosynthesis [9,10]. However, it is known that plants can take up bicarbonate ion (HCO_3_^−^) from the soil solution through their roots [33]. This process is passive and depends on the rate of transpiration, and the HCO_3_^−^ content decreases with distance from the root [34]. Zamanian et al. [17] identified the origin of C in CaCO_3_ of *B. arvensis* fruits and concluded that only about 6% of fruit carbonate originated from soil carbonates. Thus, it can be assumed that the carbonates of *B. arvensis* fruits analyzed in this study contain predominantly atmospheric carbon assimilated through photosynthesis.

Despite the lime-mediated increase in soil Si, the content of silica structures, or phytoliths, in *B. arvensis* nutlets decreased with an increase in the rate of CaCO_3_ application to the soil. This could be due to increased calcium precipitation and enhanced carbonate formation. It can be assumed that in alkaline soils with high calcium availability, calcite formation is preferential to silica formation, whereas in acidic soils, calcite deposition decreases, which is offset by increased silica precipitation. Thus, soil acidity influenced the ratio of phytoliths to calcite in plant fruits, with this ratio shifting downward as soil pH increased. However, the physiological mechanisms of this phenomenon require further study.

Interestingly, the content of PhytOC tended to increase as the content of phytoliths in the fruits decreased (Figure 3a,b). Although this increase was not monotonous across the range of calcium application rates studied. The increase in PhytOC content could be due to the fact that the less Si is precipitated in the plant structure, the more carbon-containing organic material remains occluded in the mineral phase. Furthermore, Hodson [14] reported two fractions of phytoliths differing in density, with the light fraction of phytoliths containing more than three times more carbon than the heavy fraction. It can be assumed that the increase in PhytOC with a decrease in the amount of phytoliths in *B. arvensis* fruits may be associated with the redistribution of phytolith fractions due to changes in soil acidity and Ca content, with an increase in the amount of the light fraction relative to the heavy fraction under increased soil pH. This assumption is purely speculative, so further research is needed to assess the fractional composition of phytoliths and their modification in the changing environment.

Phylogenetic differences in plant silicon (Si) requirements determine differences in phytolith content across plant species [35]. Furthermore, differences can manifest themselves at the organ level [21]. Variability in phytolith content may also be influenced by environmental Variation in Si availability [36]. A strong positive correlation between the content of phytoliths and Si in plant tissues has been previously revealed [37,38]; however, our study showed a negative relationship between the content of phytoliths in *B. arvensis* fruits and Si in the soil. The content of phytoliths in the dry matter of a plant varies from less than 0.5% in most dicotyledonous plants to 15% in some parts of cereal shoots [37]. The content of phytoliths in plants of meadows and arable lands can reach 2.6 and 5.8%, respectively [16], and 16% of phytoliths are formed in bamboo forests [39]. Li et al. [20] showed that riparian plants, shallow emergent plants, and floating aquatic plants produced 4.9, 4.1, and 2.0% phytoliths of plant dry weight, respectively. On average, at all levels of soil acidity, about 13% phytoliths were formed in the pericarps of *B. arvensis* plants, which is close to the phytolith content in bamboo and cereals.

The inclusion of cellulose and lignin during phytolith formation may control the carbon content of phytoliths [16]. Phytoliths of different plant species and tissues formed under various soil geochemical conditions may demonstrate variability in phytolith elemental composition, including organic carbon content [40]. Several studies have shown that phytoliths may occlude carbon in the range of 0.2 to 5.8% [16]. Among 18 wetland species, PhytOC contents ranged from 1.4 to 1.6% [20]. On average across all treatments, *B. arvensis* fruit phytoliths contained 1.4% organic carbon. Liming of soil from acidic to slightly alkaline increased the carbon content in phytoliths of *B. arvensis* fruits from 1.0% in 0 Ca plants to 1.8% in 7.5 Ca plants (Figure 3b). The reasons for the decrease in phytolith carbon content at the highest rate (10 t h^−1^) of CaCO_3_ application used in this study, compared to 7.5 t h^−1^, remain unclear and require further research. Despite the decrease in phytolith content in *B. arvensis* fruits with increasing soil Ca levels, the PhytOC per unit mass of fruits, per plant, or per hectare tended to increase (Figure 4a–c), apparently due to the increase in its share in phytoliths and also due to the increase in fruit yield caused by CaCO_3_ application.

Since almost all plant biomass carbon comes from photosynthesized atmospheric CO_2_, and given the significant correlation between the carbon isotope composition of PhytOC and plants, it is assumed that the main source of carbon occluded by phytoliths is atmospheric CO_2_ [16,41]. It has been suggested that the carbon in phytoliths may be “older” than the carbon in the plants that formed the minerals [42]. This means that plants should use soil carbon to create phytoliths and atmospheric carbon to create other plant structures. However, as noted by Hodson [41], the physiological mechanisms underlying this statement have not been explained.

Since the availability of essential macronutrients, including calcium, is reduced in acidic soils [43], we hypothesized that *B. arvensis* would not be able to actively mineralize pericarp to form calcium carbonates when grown on lime-free soil. However, the study showed that regardless of soil pH, the pericarp of plants was fully mineralized, and in the pericarps of plants grown on lime-free soil, the reduction in calcium carbonate formation was partially compensated by the formation of amorphous silica. Although CaCO_3_ application reduced the phytolith content in *B. arvensis* fruits, it increased the content of PhytOC. Taking into account the phytolith and PhytOC content, calculations showed lime-mediated increase in PhytOC content in fruit mass from 1.5 g kg^−1^ up to 2.0 g kg^−1^ in Ca plants, respectively. Taken together, these results and the data demonstrating Ca-induced increases in fruit calcium carbonate content and potential increase in yield suggest that *B. arvensis* responded to increased soil calcium by increasing carbon accumulation in biominerals formed in fruit pericarps.

## 4. Materials and Methods

### 4.1. Soil Treatment and Plant Sowing

The study was conducted in the experimental fields of the Agrobiological Station of the Karelian Scientific Center of the RAS, Petrozavodsk, Russia (61.751325, 34.346331). The climate of the area is moderately continental with maritime characteristics. The mean annual precipitation and mean annual temperature are about 700 mm and 3.8 °C, respectively. The soil in the study area was sandy loam. For the soil, silt (0.002–0.05 mm) and sand (0.05–2.0) had the highest particle size fraction, followed by clay (<0.002 mm) (Table 5). The total carbon content in the soil was 5.3%, and total nitrogen and phosphorus were 0.25 and 0.053%, accordingly. The soil had 46 mg kg^−1^ of available phosphorus and 34 mg kg^−1^ of available potassium. The soil was acidic with pH values ranging from 4 to 5.

In August 2024, we prepared 15 plots of 2.0 m × 1.0 m, arranged in three rows of five plots. The distance between plots and the width of the edge strip were 0.5 m. CaCO_3_ (Manufacture ‘Starorusskaja sel’hozchimia’, Staraja Russa, Russia, GOST 14050-93, total CaCO_3_ content is not less than 80%) was added at a rate of 0.0, 2.5, 5.0, 7.5, and 10.0 t ha^−1^ at a depth of 0–20 cm. The concentrations were selected during a preliminary experiment conducted in 2022–2023. The treatments were repeated three times using a block design and designated as 0 Ca, 2.5 Ca, 5 Ca, 7.5 Ca, and 10 Ca, respectively.

In September 2024, seeds of *B. arvensis* were sown in all prepared plots at a rate of 200 seeds per m^2^. In natural conditions, *B. arvensis* growth density varies greatly, from solitary individuals to densely populated populations. In this study, we selected a planting density that prevented competition for light. No winter seed protection or additional plant irrigation was provided. In May–June 2025, weeds, excluding *B. arvensis*, were removed twice from the plots, as well as between plots and along the edges.

### 4.2. Plant Growth and Productivity

From April to August 2025, shoot development was monitored, and the stages of germination, flowering, and fruit ripening were recorded. The number of seedlings in each experimental plot was determined. In early July, when the plants had accumulated their maximum biomass, ten plants were selected from each plot to determine shoot height and weight, as well as the number of inflorescences, fruits, and nutlets in the fruits. In early August, when the plants had shed their leaves, changed color, and the stems had dried out, the fruit harvest was collected. The nutlets were air-dried for 30 days, and the weight of 1000 nutlets was determined in four replicates. The pericarp of 100 nutlets of each treatment was separated, and the weight was determined per 100 nutlets in three replicates. Fruit yield per plant was calculated as the number of nutlets per plant multiplied by their average weight. We did not observe any cases of plant death, and the density of plants that reached maturity was equal to the number of seedlings. Fruit yield per unit area was calculated as fruit yield per plant multiplied by the number of plants per unit area. It should be noted that area-based yields are closely related to the planting density chosen for this study and may differ from the fruit yield of *B. arvensis* under natural plant growth conditions.

### 4.3. Plant Physiological Parameters

In early July, CO_2_ gas exchange parameters were measured on six fully expanded leaves of *B. arvensis*, randomly selected for each plot, using a portable photosynthesis system (HCM-1000, Walz, Effeltrich, Germany) at the leaf chamber temperature of 20 °C, air humidity of 60–70%, the flow rate of 600 mL/min, and the ambient CO_2_ concentration of 400 ± 20 ppm. The leaf area-based net CO_2_ assimilation rate (*A*_n_), transpiration (Tr) rate, stomata conductance (*g*_s_), and the ratio of intercellular (*C*_i_) to ambient (*C*_a_) CO_2_ concentration were determined starting at 1000 μm m^−2^ s^−1^ photosynthetic photon flux density (PPFD). The rate of leaf respiration (*R*) was taken after 30 min of 0 μm m^−2^ s^−1^ PPFD.

### 4.4. Scanning Electron Microscopy

Ten nutlets from each plot were selected to create cross-sections for scanning electron microscopy (SEM). To achieve this, the nutlets were embedded into epoxy resin, and after hardening, they were cut in the direction from the top to the cicatrix, ground, and polished. For imaging and energy dispersive X-ray (EDX) analysis, we used SEM VEGA II LSH (Teskan, Brno-Kohoutovice, Czech Republic) connected with energy dispersive microanalyzer INCA Energy 350 (Oxford Instruments, Abingdon, Oxfordshire, UK). Preliminarily, the nutlet cross-sections were attached to aluminum stubs using double-sided adhesive tape and sputter-coated with a gold layer of 10–15 nm thickness. To detect images of nutlets and pericarp cross-sections, the SEM was equipped with a secondary electron detector. To generate color element-mapping images, a backscattered electron (BSE) detector was used. EDX analyses were conducted with a W cathode, accelerating voltage of 20 kV, beam current of 20 nA, and beam diameter of 2 μm. The mineral composition was identified by the point and area SEM analysis.

### 4.5. Nutlet Morphology Parameters

The SEM images of nutlet cross-sections were used to determine the morphological characteristics of the nutlets. The length and width of the nutlets, as well as the thickness of the pericarp in the main part and cicatrix, were measured using the software package ImageJ (ImageJ 1.54k for Windows).

### 4.6. Soil Chemical Analyses

For chemical analyses, soil samples were collected from a depth of 0–20 cm. The chemical analyses of soil from each plot and plant samples were carried out in the Core Facility “Analytical laboratory” of the Forest Institute of KRC of RAS. To measure soil pH, soil samples were air-dried and sieved with a 2.0 mm sieve. The pH_H2O_ of each soil sample was measured in water at a ratio of 1:25 (*w*:*v*), and pH_KCl_ was recorded in 1 M KCl solution at a ratio of 1:25 (*w*:*v*) using a HI 2210 pH-meter (Hanna GmbH, Vöhringen, Germany). Before chemical analysis, samples were ground in a laboratory mill and sieved with a 0.05 mm sieve. Crushing and sifting the soil allowed for a more thorough separation of plant remains, as well as increased accuracy in determining the content of the elements being studied. Total soil C content was determined by the spectrophotometric method of potassium dichromate oxidation using an SF2000 spectrophotometer (Spektr, Sankt-Petersburg, Russia). The total N content in the soil was determined spectrophotometrically using the Kjeldahl method. Total soil P was measured spectrophotometrically using a modified Denigès-Atkins molybdate method [44]. The content of available P in soil extracts (0.2 M HCl) was measured using the ammonium molybdate spectrophotometric method (SF2000, Spektr, Sankt-Petersburg, Russia). Available K, Ca, and Si in the soil were extracted with 0.2 N HCl. K content was determined by the emission method, and Ca content was recorded by the flame atomization method using an AA-7000 spectrophotometer (Shimadzu, Kyoto, Japan). Soil available Si was measured by electrothermal atomization with palladium nitrate matrix modifier using an atomic AA-6800 spectrophotometer (Shimadzu, Kyoto, Japan).

### 4.7. Measurement of Carbonates, Phytoliths, and PhytOC Content

Phytoliths were isolated from nutlets of each treatment using the dry aching extraction method with minor modifications [45,46,47] in the Core Facility “Analytical laboratory” of the Forest Institute of KRC of RAS. For that, 1 g of plant material was placed in platinum crucibles with platinum lids and processed in a muffle furnace at 550 °C for 4 h. The temperature was raised gradually by 50 °C to prevent the loss of plant material. After cooling, the ash content was calculated. Then the crucibles were placed on hotplates, and 5 mL 5% hydrochloric acid was added twice to separate the acid-soluble carbonate fraction. The content of the carbonate fraction was calculated as the difference in sample weight before and after acid treatment. We assume that in our case, the acid-soluble carbonate fraction largely reflects the carbonate content in the fruits of the studied species. The carbon content of the carbonate fraction was calculated as the proportion of C in CaCO_3_.

After cooling, the resulting residue, designated phytolith, was then washed with distilled water and dried at 105 °C to constant weight and weighed. The carbon content in phytolith was measured using absolutely dry material on a Unicube Elementar elemental analyzer (Elementar GmbH, Langenselbold, Germany), calibrated with a certified acetanilide standard. All fractions were calculated as a percentage of the initial sample gravimetrically (Sartorius CP124S, Göttingen, Germany).

### 4.8. Statistical Test

All data are shown as means ± SE values. All statistical tests were performed using Statistica software (version 8.0.550.0, StatSoft, Inc., Tulsa, OK, USA). For each plot, the parameters studied were averaged to obtain a single independent data point, which was used for a statistical test. For all means, *n* = 3. To assess significant differences between the treatments, the least significant difference (LSD) test of analysis of variance (ANOVA) was used at a significance level of *p* < 0.05.

## 5. Conclusions

This study examined the carbon sequestration potential of biominerals formed in the fruits of *B. arvensis*, a member of the Boraginaceae family. Biomineralization occurred throughout the fruit pericarp with the formation of silica and calcium carbonate, regardless of the calcium content in the soil and soil pH. However, with increasing calcium availability, the silica to carbonate ratio changed towards a decrease in silica content and an increase in carbonate fraction content. Increased soil Ca content caused an increase in the amount of silica-occluded organic carbon and carbonate carbon in *B. arvensis* fruits, which, combined with increased fruit yield, contributed to more efficient carbon accumulation in biominerals. Using the example of the growth of this weedy species in conditions of different calcium content in the soil, the possibility of variability in the characteristics of mineral formation in plants under the influence of environmental conditions was confirmed. However, further long-term studies of plants growing in different climatic conditions are needed to more fully identify patterns of mineralization and carbon accumulation in biominerals.

## Figures and Tables

**Table 1:** Physicochemical properties of soil after the application of CaCO_3_ at a rate of 0.0 (0 Ca), 2.5 (2.5 Ca), 5.0 (5 Ca), 7.5 (7.5 Ca), and 10.0 (10 Ca) t ha^−1^.

Parameter	0 Ca	2.5 Ca	5 Ca	7.5 Ca	10 Ca
pH_H2O_	5.36 ± 0.01 e*	6.09 ± 0.01 d	6.23 ± 0.02 c	6.49 ± 0.03 b	7.33 ± 0.02 a
pH_KCl_	4.46 ± 0.01 e	5.15 ± 0.03 d	5.21 ± 0.02 c	5.66 ± 0.01 b	6.94 ± 0.01 a
Total C, g kg^−1^	53 ± 4 b	49 ± 4 bc	80 ± 4 a	44 ± 1 c	41 ± 1 c
Total N, g kg^−1^	2.5 ± 0.2 b	2.5 ± 0.2 b	4.6 ± 0.1 a	2.7 ± 0.4 b	2.4 ± 0.1 b
Total P, g kg^−1^	0.53 ± 0.02 a	0.51 ± 0.01 ab	0.47 ± 0.01 bc	0.47 ± 0.01 bc	0.46 ± 0.01 c
Available P, mg kg^−1^	46 ± 1 a	45 ± 2 a	45 ± 1 a	39 ± 2 b	44 ± 1 ab
Available K, mg kg^−1^	34 ± 0 b	37 ± 2 a	39 ± 0 a	38 ± 1 a	38 ± 1 a
Available Ca, g kg^−1^	1.6 ± 0.1 d	2.2 ± 0.2 bc	1.8 ± 0.1 cd	2.4 ± 0.1 b	4.1 ± 0.1 a
Available Si, mg kg^−1^	137 ± 4 d	139 ± 7 d	183 ± 9 c	205 ± 1 b	237 ± 6 a

* Different letters indicate significant differences between the means.

**Table 2:** Growth and yield characteristics of *Buglossoides arvensis* grown on soil treated with CaCO_3_ at concentrations of 0.0 (0 Ca), 2.5 (2.5 Ca), 5.0 (5 Ca), 7.5 (7.5 Ca), and 10.0 (10 Ca) t ha^−1^.

Parameter	0 Ca	2.5 Ca	5 Ca	7.5 Ca	10 Ca
Seeding density, plant m^−2^	58 ± 6 b*	97 ± 10 a	116 ± 13 a	122 ± 12 a	116 ± 11 a
Plant height, cm	55 ± 2 c	58 ± 2 bc	64 ± 2 ab	69 ± 3 a	63 ± 4 ab
Shoot dry weight, g	1.3 ± 0.1 c	1.5 ± 0.1 b	1.9 ± 0.2 a	1.8 ± 0.2 ab	1.7 ± 0.2 abc
Nutlet weight, g per 1000 nutlets	2.79 ± 0.02 c	2.93 ± 0.02 b	2.91 ± 0.03 b	2.91 ± 0.02 b	3.02 ± 0.02 a
Pericarp weight, g per 1000 nutlets	1.87 ± 0.03 a	1.97 ± 0.10 a	1.93 ± 0.02 a	1.98 ± 0.09 a	1.91 ± 0.09 a
Pericarp weight, % nutlet	67	67	66	68	63
Number of inflorescences per plant	3.7 ± 0.3 a	3.1 ± 0.2 a	3.5 ± 0.3 a	3.4 ± 0.2 a	3.1 ± 0.2 a
Number of fruits per plant	47 ± 5 b	46 ± 3 b	58 ± 5 ab	63 ± 6 a	52 ± 5 ab
Number of nutlets per plant	187 ± 20 b	185 ± 13 b	232 ± 22 ab	253 ± 26 a	207 ± 20 ab
Yield, g plant^−1^	0.52 ± 0.05 b	0.54 ± 0.04 b	0.67 ± 0.06 ab	0.74 ± 0.08 a	0.63 ± 0.06 ab
Yield, t ha^−1^	0.30 ± 0.03 c	0.52 ± 0.06 b	0.78 ± 0.08 a	0.90 ± 0.08 a	0.73 ± 0.07 ab

* Different letters indicate significant differences between the means.

**Table 3:** Physiological traits of *Buglossoides arvensis* grown on soil treated with CaCO_3_ at concentrations of 0.0 (0 Ca), 2.5 (2.5 Ca), 5.0 (5 Ca), 7.5 (7.5 Ca), and 10.0 (10 Ca) t ha^−1^.

Parameter	0 Ca	2.5 Ca	5 Ca	7.5 Ca	10 Ca
*A*_n_ *, μmol m^−2^ s^−1^	8.8 ± 0.7 a	10.0 ± 0.8 a	8.0 ± 0.6 a	8.9 ± 0.4 a	7.9 ± 0.7 a
Tr, mmol m^−2^ s^−1^	1.38 ± 0.10 a	1.50 ± 0.16 a	1.29 ± 0.08 a	1.30 ± 0.09 a	1.24 ± 0.06 a
*g*_s_, mmol m^−2^ s^−1^	112 ± 13 a	134 ± 20 a	109 ± 16 a	102 ± 9 a	96 ± 8 a
*C*_i_:*C*_a_	0.58 ± 0.03 a	0.54 ± 0.04 a	0.57 ± 0.05 a	0.57 ± 0.03 a	0.52 ± 0.05 a
*R*, μmol m^−2^ s^−1^	0.72 ± 0.03 a	0.70 ± 0.05 a	0.69 ± 0.06 a	0.64 ± 0.04 a	0.65 ± 0.10 a

* *A*_n_, net CO_2_ assimilation rate; Tr, transpiration rate; *g*_s_, stomatal conductance; *C*_i_:*C*_a_, the ratio of intercellular to ambient CO_2_ concentration; *R*, leaf respiration. Different letters indicate significant differences between the means.

**Table 4:** Morphological characteristics of nutlets of *Buglossoides arvensis* grown on soil treated with CaCO_3_ at concentrations of 0.0 (0 Ca), 2.5 (2.5 Ca), 5.0 (5 Ca), 7.5 (7.5 Ca), and 10.0 (10 Ca) t ha^−1^.

Parameter	0 Ca	2.5 Ca	5 Ca	7.5 Ca	10 Ca
Nutlet height, mm	2.13 ± 0.02 b*	2.19 ± 0.02 a	2.16 ± 0.02 ab	2.15 ± 0.02 ab	2.13 ± 0.02 b
Nutlet width, mm	1.50 ± 0.02 a	1.54 ± 0.02 a	1.49 ± 0.02 a	1.50 ± 0.01 a	1.49 ± 0.02 a
Pericarp thickness mean, μm	100 ± 3 b	117 ± 5 a	110 ± 4 ab	101 ± 4 b	115 ± 4 a
Cicatrix thickness, μm	115 ± 9 a	110 ± 7 a	111 ± 4 a	98 ± 7 a	105 ± 4 a

* Different letters indicate significant differences between the means.

**Table 5:** Particle size distribution of the soil in the study area.

Particle size, mm	<0.002	0.002–0.05	0.05–2.0	>2.0
Distribution, %	4.1	48.2	47.8	0.0

## Data Availability

The original contributions presented in this study are included in the article. Further inquiries can be directed to the corresponding author.

## Abbreviations

The following abbreviations are used in this manuscript:

EDX	Energy dispersive X-ray
PhytOC	Phytolith-occluded carbon
SEM	Scanning electron microscopy
